# Cytokeratin 8 ectoplasmic domain binds urokinase-type plasminogen activator to breast tumor cells and modulates their adhesion, growth and invasiveness

**DOI:** 10.1186/1476-4598-8-88

**Published:** 2009-10-21

**Authors:** Nataša Obermajer, Bojan Doljak, Janko Kos

**Affiliations:** 1 Jožef Stefan Institute, Department of Biotechnology, Jamova 39, 1000 Ljubljana, Slovenia; 2 University of Ljubljana, Faculty of Pharmacy, Aškerèeva 7, 1000 Ljubljana, Slovenia

## Abstract

**Background:**

Generation of plasmin is a characteristic of tumor cells, promoting the degradation of extracellular matrix, tumor progression and metastasis. The process is accelerated if plasminogen and plasminogen activator are bound to their cell surface receptors.

**Results:**

In this study we show that the monoclonal antibody that recognizes an epitope on the cytokeratin 8 (CK8) ectoplasmic domain (anti-CK MAb) inhibits plasminogen activation mediated by urokinase-type plasminogen activator (uPA) in MCF-7 and MCF-10A neoT cells. The ectoplasmic domain of CK8 acts as a binding site for plasminogen, however, by using confocal microscopy, we demonstrated that it is also co-localized with uPA. CK8, therefore, function also as a receptor for uPA on the cell surface, and the presence of anti-CK MAb may prevent the binding of uPA to a designated CK8 motif. The consequent inhibition of plasmin generation resulted in changed cell morphology, enhanced cell adhesion to fibronectin, reduced invasion potential, and an enhanced G1/S transition. Moreover, surface plasmon resonance analysis showed that the synthetic dodecapeptide corresponding to the epitope sequence (VKIALEVEIATY), binds uPA in the nanomolar range.

**Conclusion:**

These novel findings suggest a model in which CK8, together with uPA, plasminogen and fibronectin, constitutes a signaling platform capable of modulating cell adhesion/growth-dependent signal transduction in breast tumor cells. Anti-CK MAb, which competes for the binding site for uPA, could be used as an agent to reduce the invasive potential of breast tumor cells.

## Background

Proteases are essential for invasion by tumor cells. For this, they need to be activated, either on the tumor cell surface or in the pericellular space. In breast cancer, urokinase type plasminogen activator (uPA), a serine proteinase, has been associated with an aggressive malignant phenotype [1,2]. Cell surface uPA, bound to uPA receptor (uPAR), activates plasminogen to plasmin, a central player in breast cancer progression and metastasis. Plasmin activates growth factors and protease cascades that lead to the disruption of cell-cell and cell-extracellular matrix adhesion via pericellular proteolysis of glycoproteins [3,4]. Plasmin can also affect the cell phenotype by activating or inactivating growth factors, and by modifying growth factor receptors and adhesion receptors [5-7].

Plasminogen is activated on the cell surface much faster than in solution, due to the presence of proteins that promote a plasminogen conformation more open to proteolytic attack [8]. In addition to uPAR, cytokeratin 8 (CK8) is an important plasminogen-binding protein in the membrane of breast cancer cells. CK8 is an intermediate filament protein and associates with CK18 to form an insoluble matrix within the cell. However, the C-terminal end of CK8 also penetrates the cellular membrane, as shown for hepatocellular and breast carcinoma cells [9,10]. CK8 is unique among cytokeratins in that it contains a carboxyl-terminal lysine that can interact with the lysine binding sites of plasminogen, although it is able to bind plasminogen even when the C-terminal lysine is mutated [11]. Plasminogen activation is promoted by tissue type plasminogen activator (tPA), which can bind to both components of the cytokeratin heterodimer, CK8/CK18. Plasminogen and tPA cannot bind to CK8 at the same time, but the binding of tPA to CK18 enhances the binding of plasminogen to CK8 and its activation [11].

The CK8/CK18 complex modulates the signaling pathways intracellularly by binding kinases involved in signal transduction. It integrates signals generated by stimulated surface membrane receptors, such as insulin receptor and integrin beta 1 receptor, and modulates their signal transduction in appropriate reaction sequences. The absence of CK8/CK18 in hepatocytes results in a phenotype that attaches more rapidly to fibronectin and undergoes an enhanced G1/S transition compared with wild-type hepatocytes [12]. On the other hand, the cytoskeleton itself can undergo rearrangements as a result of outside-in signals triggered by fibronectin binding to cell surface receptors, the integrins.

The aim of this study was to investigate the involvement of CK8 in plasminogen activation, and consequently in cell adhesion, invasion and signaling of breast tumor cells. An anti-cytokeratin MAb directed against an epitope on the ectoplasmic tail of CK8 was used to determine whether it prevents uPA mediated activation of plasminogen, resulting in enhanced adhesion of breast tumor cells to fibronectin, reduced invasion, as well as changed morphology and enhanced G1/S transition.

## Results

### Plasminogen expression in breast tumor cells

**Figure 1:**
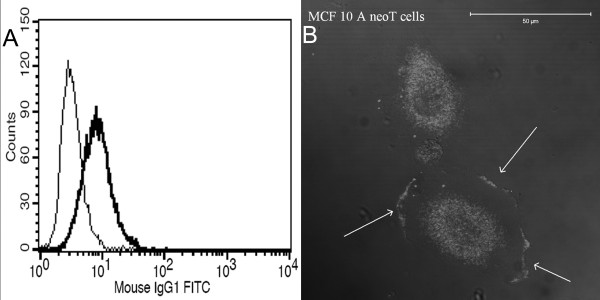
**Plasminogen expression and localization in MCF10A neoT cells**. (**A**) Flow cytometry histogram of MCF-10A neoT cells stained with anti-plasminogen antibody. Surface expression of plasminogen was evaluated in MCF10A neoT cells cultured on fibronectin in serum-free medium (bold line). The thin line represents the isotype control. (**B**) Plasminogen localization in MCF-10A neoT cells. Non-permeabilized breast cancer cells cultured on fibronectin-coated glass coverslips were stained with anti-plasminogen antibody. Plasminogen is distributed on the surface of cells (pointing arrows).

Plasminogen was detected by flow cytometry in MCF-10 A neoT cells that were cultured in serum-free medium (Figure 1A). Staining with anti-plasminogen antibody showed the presence of plasminogen on the plasma membrane in non-permeabilized MCF-10 A neoT cells (Figure 1B). The staining pattern of plasminogen on the plasma membrane of non-permeabilized MCF cells was shown, by confocal microscopy, to be similar to that obtained with anti-CK MAb [13]. Observation of several confocal planes confirms that the staining follows the surface of the cell.

### uPA localization in breast tumor cells

**Figure 2:**
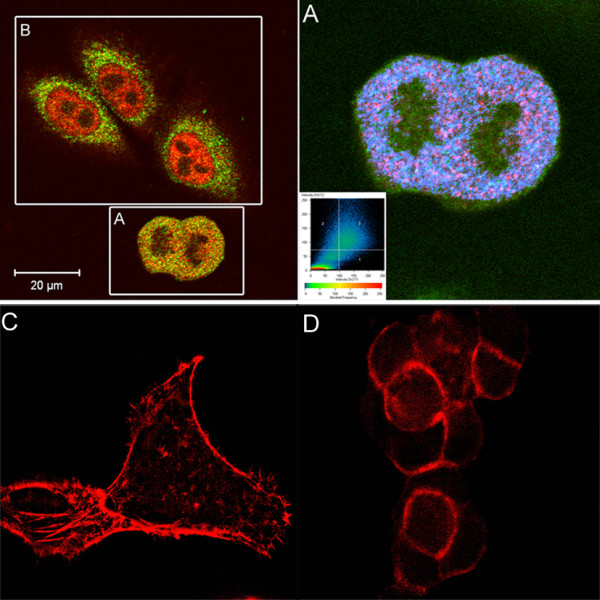
**Localization of uPA, cytokeratin and actin in MCF10A neoT cells**. Colocalization of anti-uPA antibody and anti-CK MAb (**A**). MCF-10A neoT cells were stained with anti-uPA MAb (green fluorescence, Alexa-488) and anti-CK MAb (red fluorescence, Alexa-555) and imaged sequentially in a line-interlace mode to eliminate cross talk between the channels. The threshold level for this display was set arbitrarily. Pixels above the threshold in both channels (blue color, right panel) and the contour plot are shown for MCF10A neoT cell cluster (inset C) demonstrating strong co-localization in contrast to adherent MCF-10A neoT cells (**B**). Anti-CK Mab triggered actin reorganization in MCF-10A neoT cells. MCF-10A neoT cells, treated (**C**) and non-treated (**D**) with anti-CK MAb, were cultured on fibronectin for 2 h and stained with phalloidin. Actin bundles are seen in anti-CK8 MAb treated cells but not in non-treated cells.

The treatment of MCF10A neoT cells with anti-CK MAb markedly affected breast tumor cell morphology. Whereas non-treated cells, grown on a fibronectin-coated surface, did not form typical epitheloid morphology but floated in clusters in the medium, anti-CK MAb treated breast tumor cells spread over the fibronectin-coated surface accompanied by rearrangement of the cytoskeleton, as evident by the reorganization of actin in bundles (Figures 2A and 2B).

uPA was co-localized with cytokeratins, detected with anti-CK MAb, in MCF-10A neoT cells cultured on fibronectin, which were not spread on the substratum but loosely attached in a cell cluster. On the other hand, uPa and cytokeratins were not co-localized in MCF-10A neoT cells that were firmly attached to the substratum and that exhibited epitheloid morphology (Figures 2C and 2D).

### Binding of uPA to a cytokeratin sequence recognized by anti-CK MAb

**Figure 3:**
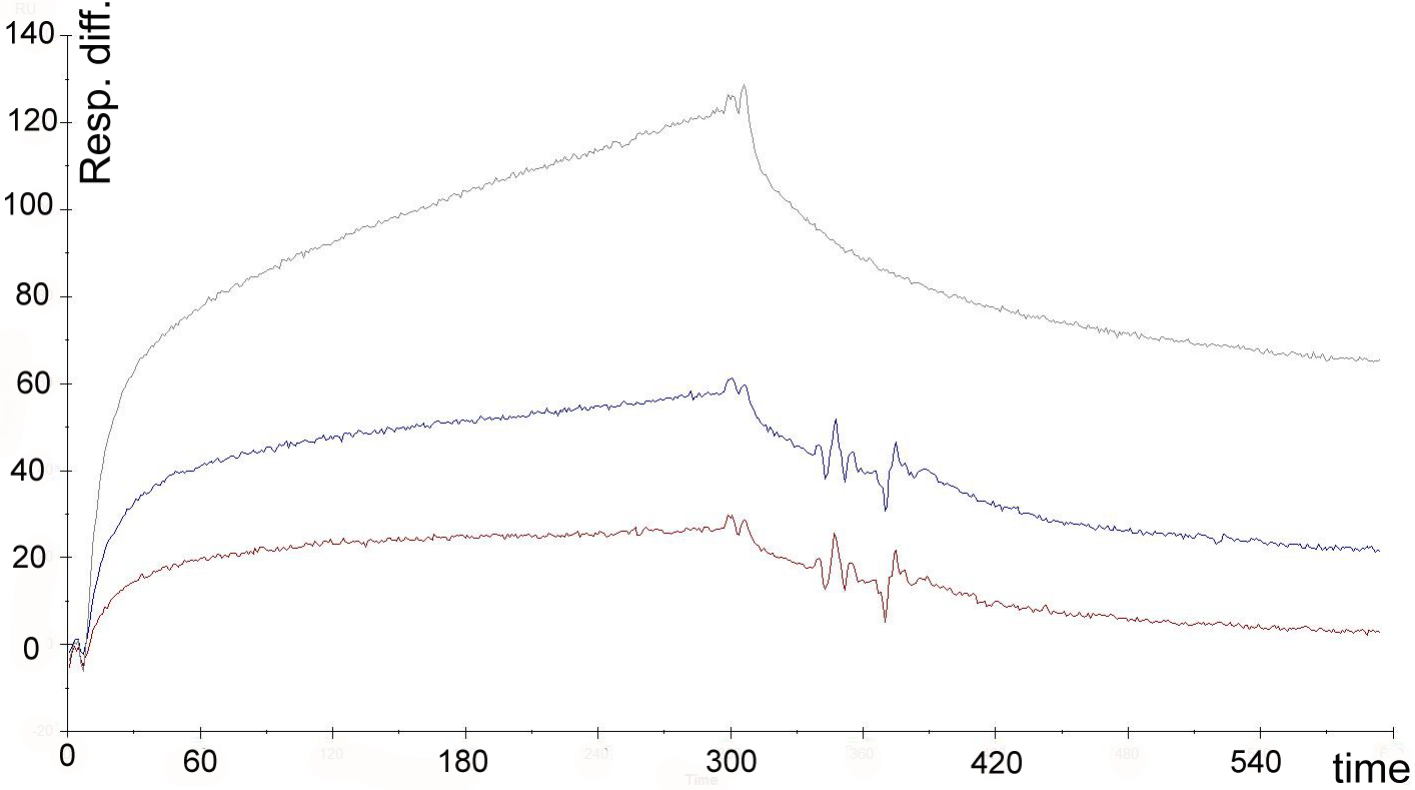
**uPA binding to dodecapeptide VKIALEVEIATY**. Association and dissociation curves of uPA binding to VKIALEVEIATY peptide, obtained by surface plasmon resonance (from the bottom up, uPA at 10 nM, 20 nM and 50 nM). A *K*_a _of 9.8 nM was calculated, using the 1:1 binding with drifting baseline model.

The binding of uPA and plasminogen to the peptide VKIALEVEIATY, deduced from the C-terminal region of CK1, CK2, CK8, VK10, and recognized by anti-CK MAb [13], was assessed by surface plasmon resonance analysis. While the binding of plasminogen to the surface with immobilized peptide was not significantly different from that to the control flow cell surface lacking the peptide (data not shown), uPA bound specifically to the peptide in a concentration dependent manner, with a *K*_a _of 9.8 nM (Figure 3).

Additionally, the binding of uPA and anti-CK MAb was tested simultaneously in ELISA on immobilized peptide VKIALEVEIATY. The binding of anti-CK MAb was followed by the secondary anti-mouse antibody, conjugated to horse radish peroxidase or fluorescence dye Alexa Fluor 488. uPA and MAb competed for the binding to the peptide in a dose response manner (see additional file 1).

### Adhesion of breast tumor cells to fibronectin is increased by anti-CK MAb

**Figure 4:**
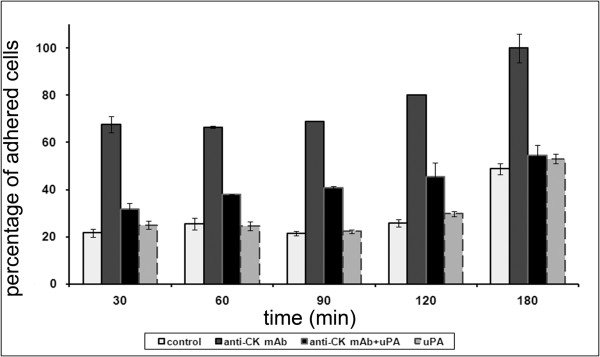
**Adhesion of MCF-10A neoT cells to fibronectin in the presence of anti-CK MAb and uPA**. MCF10A neoT cells were cultured on a fibronectin coated surface in the absence or presence of anti-CK MAb and/or uPA. The percentage of adherent cells was assessed with MTS assay. The time course of adhesion to the fibronectin surface is shown (See also Video S1 and S2). Samples were analyzed in quadruplicate and each point is the mean of at least three independent experiments.

**Figure 5:**
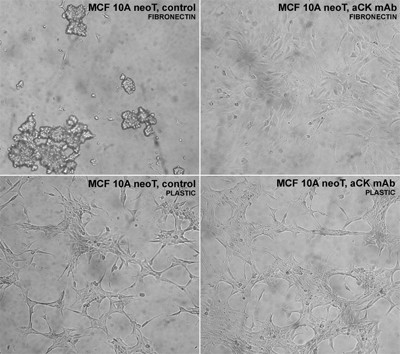
**Anti-CK MAb changes the morphology of MCF-10A neoT cells cultured on fibronectin**. Photomicrographs of MCF-10A neoT cells cultured for 24 h on uncoated (below) or fibronectin-coated surfaces (above) are shown for control and anti-CK8 MAb treated cells. Anti-CK MAb enables the adhesion to fibronectin of MCF-10A neoT cells, but not control cells, which form cell clusters. In contrast, both control and anti-CK MAb treated cells adhere to uncoated surface.

When MCF-10A neoT cells were seeded on fibronectin, they adhered more rapidly and to a greater extent in the presence of anti-CK MAb than in its absence (Figure 4). For instance, at 30 min post seeding, 67.7% of MCF-10A neoT cells were already attached in the presence of the anti-CK MAb as compared to 21.7% in its absence. The fact that the effect of anti-CK MAb was evident so quickly indicates that it acts by binding to the cell surface epitope and not by its intracellular activity. Even after 200 min, only 48.8% of untreated cells were attached (Additional File 2), while anti-CK MAb treated MCF-10A neoT cells were all attached (Additional File 3). However, when uPA was added to the culture medium, the anti-CK MAb-stimulated adhesion of MCF-10A neoT cells was diminished (54.5% after 200 min), demonstrating that increased plasmin activity mediated by uPA reduces the adhesion of breast tumor cells to fibronectin. Anti-CK MAb did not alter cell adhesion to an uncoated surface, showing the substrate-dependent effect of the MAb (Figure 5).

### Invasion

**Figure 6:**
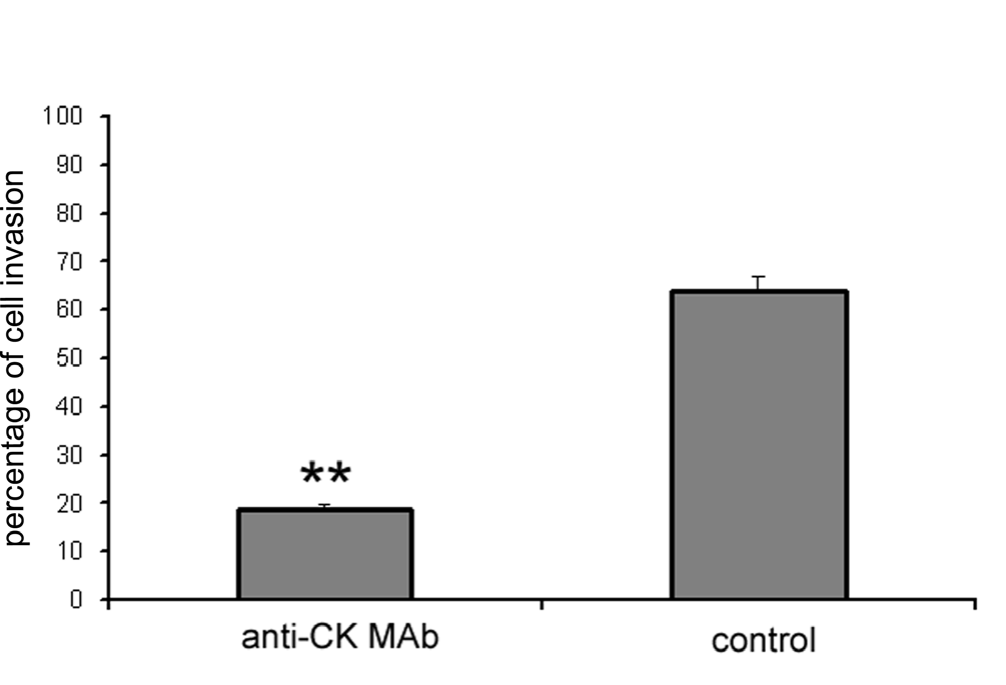
**Anti-CK MAb reduces the invasiveness of MCF-10A neoT cells**. The degree of invasion of Matrigel by MCF-10A neoT cells is shown in the presence and absence of anti-CK MAb. It is presented as the percentage of invasion of MCF-10A neoT cells to the lower compartment of a Transwell chamber. Bars represent mean percentages of migrated cells ± s.d. P values < 0.001 are marked with **.

Anti-CK MAb was tested for its effect on the invasion of breast tumor cells through Matrigel. The addition of anti-CK MAb to MCF-10A neoT cells strongly reduced the degree of invasion compared to control cells (Figure 6). This result can again be related to plasminogen activation on breast cancer cells. However, the viability and proliferation of breast tumor cells on Matrigel were not affected after 24 h by anti-CK MAb, as detected by MTS assay (data not shown).

### Effect of anti-CK MAb on the G1/S transition of breast tumor cells

**Figure 7:**
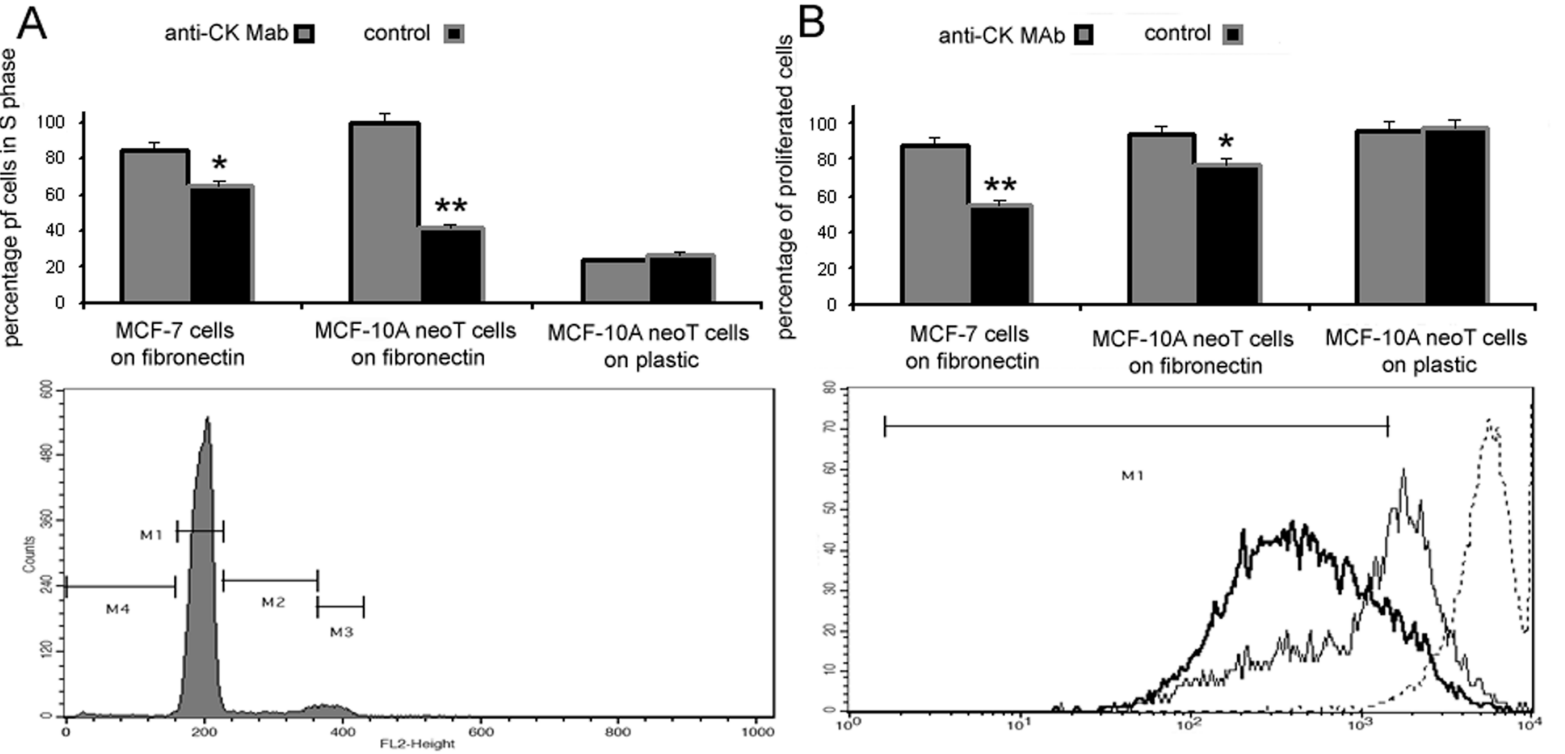
**The effect of anti-CK MAb on cell cycle (A) and proliferation (B) of breast tumor cells cultured on fibronectin**. MCF-10A neoT and MCF-7 cells were cultured in serum-free medium in the absence or presence of anti-CK MAb. After 24 h they were harvested and analyzed for cell cycle status by flow cytometry. The percentage of anti-CK MAb treated and untreated cells in S phase is given (A, top). The representative histogram used to determine the percentage of distinctive cell populations is shown at the bottom. For the proliferation assay, MCF-7 cells were labeled with CFSE reagent and cultured for 48 h in the presence or absence of anti-CK MAb. Proliferation threshold was set according to CFSE labeled cells analyzed immediately after labeling (B, bottom). The percentage of proliferating cells of the PI^neg ^population is given (B, top). Results represent at least three independent assays. P values of < 0.05 are marked with *, and P values < 0.001 with **.

Breast tumor cells need to be seeded on a substratum in order to progress through the cell cycle in response to growth stimulation. On seeding MCF neoT cells on fibronectin in the presence of anti-CK MAb, more cells entered S phase. The percentage of cells in S phase, as detected by flow cytometry, was 4.2% for control cells and 10.0% for anti-CK MAb treated cells. In contrast, the G1/S transition was not affected by anti-CK MAb when the cells were cultured on an uncoated surface (Figure 7A).

### Enhanced proliferation of breast tumor cells on fibronectin by anti-CK MAb

The extent of breast tumor cell proliferation on fibronectin in the presence or absence of anti-CK MAb was assessed using the CFSE reagent. The CFSE proliferation assay does not depend on the metabolic activity of breast tumor cells and enables detection of cells that have undergone cell division. The addition of anti-CK MAb enhanced the proliferation of cells cultured on fibronectin from 77.3% to 94.2% (MCF10A neoT cells) and from 54.8% to 87.3% (MCF-7 cells). No enhancement was observed on surfaces not covered with fibronectin (Figure 7B).

## Discussion

We have demonstrated that uPA is able to bind a particular motif on the CK8/CK18 complex expressed on the plasma membrane of breast tumor cells, that is recognized also by anti-CK MAb. The antibody hinders uPA binding to the cell surface of MCF-7 and MCF-10A neoT breast cells [13], resulting in lower plasmin generation, adhesion, invasiveness and G1/S transition of breast tumor cells.

Although plasminogen does not bind to the motif that resembles the epitope of anti-CK MAb (data not shown), anti-CK MAb significantly reduces plasminogen activation in MCF-7 and MCF-10A neoT cells [13]. Plasminogen is shown to be expressed endogenously in these cells and localized intracellularly as well as on the plasma membrane. The activation of plasminogen to plasmin enables degradation of the proteins of the extracellular matrix, a process that enhances the invasive potential of tumor cells. Plasminogen activation is enhanced if plasminogen activator (uPA/tPA) and the substrate (plasminogen) are in close juxtaposition on the cell surface. The most prominent uPA binding receptor is uPAR (CD87). However, plasminogen activation independent of uPAR suggests that other uPA binding proteins exist on the plasma membrane. On neutrophils, for example, α_M_β_2 _integrin receptor binds uPA irrespectively of the presence of uPAR [14]. In this case, uPAR may serve as an enhancer of α_M_β_2 _binding, and uPAR, uPA, α_M_β_2 _and plasminogen may form a multicontact complex on the cell surface. MCF-7 cells, for example, express very low levels of cell surface uPAR compared to the larger amount of cell surface bound plasminogen [15]. Our results show that CK8, the major plasminogen receptor on breast tumor cells [16], may also serve as a uPA binding protein and enhance uPA mediated plasminogen activation. As shown by surface plasmon resonance analysis, the interaction between uPA and CK8 is significant, since uPA binds the peptide VKIALEVEIATY, deduced from the C-terminal region of CK8, in the nanomolar range (*K*_a _of 9.8 nM). This peptide, which also binds strongly to the anti-CK MAb, contains a consensus sequence, corresponding to the most conserved region between D381 and E407 (CK8) in CK1, CK2, CK8, CK10 and CK18. Anti-CK MAb was shown to recognize these CKs, therefore, we conclude that the epitope is identical, independently of the exact amino acid sequence [13].

Expression of CK8 on the cell surface correlates with increased invasiveness *in vitro *and *in vivo *[17,18] and may predispose cell polarization and adhesion [19]. We have shown that anti-CK MAb, by diminishing the uPA mediated plasminogen activation in serum-free environment [13], affects the de-adhesion-invasion processes of breast tumor cells. The adhesion of these cells treated with anti-CK MAb was greatly enhanced, due to the reduced pericellular proteolysis of fibronectin, increasingly degraded in the tumor environment by plasmin. This is consistent with the findings of Tan et al. who showed that plasminogen expression, restricted to cells with high invasion ability, is involved in cell dissociation, the first important step in the invasion process [20]. The adhesion of MCF cells to fibronectin is mediated via β_1 _integrin receptors, which can be modified by plasmin in a way that affects the cell phenotype [7,21]. Although we cannot exclude a direct effect of plasmin on integrin chains, we suggest that the anti-adhesive effect of plasmin results from the proteolytic cleavage of fibronectin, similarly to the way that plasmin, when generated by the uPA-dependent cell surface-associated pathway, abrogates the HaCaT cell binding functions via cleavage of vitronectin and destruction of its cell binding function [22]. In contrast, anti-CK MAb did not affect cell adhesion to the surface not coated with fibronectin.

Our results further show that, in breast tumor cells cultured on fibronectin in serum free medium, anti-CK MAb reduces cell cluster formation and promotes cell polarization associated with actin reorganization. This is consistent with the observations that, in the presence of uPA or plasmin, MCF-7 breast tumor cells begin to retract and form solid tumor cell spheroids [23]. The spontaneous formation of the spheroids starts after supplementing the culture medium with an outdated source of human serum containing uPA and plasminogen [23].

Anti-CK MAb treated breast tumor cells cultured on fibronectin, but not on uncoated surface, also exhibited enhanced proliferation. The latter can be attributed to an increased mitogenic signal delivered by fibronectin, where the activating signal is mediated by β_1 _integrin receptor. The signaling reactions activated by β_1 _integrins include focal adhesion kinase (FAK)-Src and MAPK pathways, and generate sustained activation of the PI3K-Akt pathway, a property that could be important for anchorage-dependent survival of cells [24]. MCF-7 cell-fibronectin interaction induces the re-entry of MCF-7 cells into S phase, and prevents them from undergoing serum deprivation induced apoptosis [25]. Anti-CK MAb increases adhesion of breast cancer cells to fibronectin, thereby promoting their G1/S transition. These novel findings suggest a model by which CK8, along with plasminogen-*see earlier comment *and fibronectin, constitutes a signaling platform capable of modulating cell adhesion/growth-dependent signal transduction. A similar effect was observed in CK8-null hepatocytes, which attach more rapidly but spread more slowly on a fibronectin substratum. Loss of CK8/CK18 also leads to decreased size, reduced rate of protein synthesis, and an enhanced G1/S transition in hepatocytes [12]. The analogy between hepatocytes and the MCF-7/MCF-10A neoT used in our experiments is not surprising, considering that CK8-mediated plasmin activation has been reported on hepatocytes and hepatocellular carcinoma cells, as well as on certain breast tumor cells [19].

## Conclusion

Anti-CK MAb, that recognizes the epitope on the ectoplasmic tail of CK8 present on the plasma membrane of the breast tumor cells, prevents the binding of uPA to cytokeratin and, consequently, the activation of plasminogen to plasmin. Anti-CK MAb could thus be used as an agent to reduce the invasive potential of breast tumor cells, whereas the synthetic peptide, resembling the epitope, may regulate uPA activity.

## Methods

### Cell culture

MCF-10A neoT cells were provided by Prof. Bonnie F. Sloane (Wayne State University, Detroit, MI). The original human breast epithelial cell line (MCF-10) was transformed with neomycin resistance gene and c-Ha-ras oncogene, to give a highly invasive cell phenotype. MCF-7 cells were obtained from ATCC (HTB 22). Cells were cultured in monolayers to 80% confluence in DMEM/F12 medium supplemented with 12.5 mM HEPES, 2 mM glutamine, 5% fetal bovine serum (FCS), insulin, hydrocortisone, epidermal growth factor and antibiotics at 37°C in a humidified atmosphere containing 5% CO_2_. The cells were maintained in serum-free medium for 24 h before being used in assays. Cells were washed with PBS, pH 7.4 and detached from culture flasks with 0.05% trypsin and 0.02% EDTA in PBS, pH 7.4. The viability of cells in the experiments was at least 90%, as determined by staining with nigrosin.

### Antibody preparation

Anti-CK MAb, a mouse monoclonal antibody, was raised against soluble membrane proteins of MCF-7 human invasive ductal breast carcinoma. Using immunocytochemical analysis, its staining was detected predominantly in primary breast carcinomas and in metastatic lymph nodes [13,26]. The antibody recognizes a specific cytokeratin profile (cytokeratins 1, 2, 8, 10, 18) in MCF-7 and MCF-10A neoT breast cancer cell lines [13], as determined by 2D electrophoresis, immunoblotting and mass spectroscopy. Hybridoma cells for antibody isolation were cultured in DMEM medium supplemented with 2 mM glutamine, 13% FCS and antibiotics, at 37°C in a humidified atmosphere containing 5% CO_2_. The antibody was isolated by affinity chromatography on Protein A Sepharose using standard procedures. On MCF-7 and MCF-10A neoT it was demonstrated that the antibody inhibited the generation of plasmin in a dose dependent manner [13].

### Immunofluorescence

For flow cytometry, MCF-10A neoT cells were grown for 24 hours in serum free medium and afterwards cultured on 12-well plates pre-coated with fibronectin (Corning Costar). Cells were harvested and fixed in 4% paraformaldehyde for 30 min, and permeabilized for 10 minutes with Triton-X 100. Non-specific staining was blocked for 30 minutes with 3% BSA in phosphate buffered saline (PBS), pH 7.4. Cells were incubated with 5 μg/mL goat anti-plasminogen polyclonal antibody in blocking buffer for 1 hour on ice. Afterwards, the cells were washed with PBS and incubated with Alexa-488 labeled anti-goat secondary antibody in blocking buffer for 1 hour on ice. Alexa-488-IgG1 was used for isotype control. Cells were washed and analyzed with FACS Calibur (Becton Dickinson, NJ).

For confocal microscopy, glass coverslips were pre-treated with 20% sulfuric acid, followed by 10 M NaOH, and washed with distilled water and ethanol before use. They were coated with 1 μg/mL fibronectin in carbonate buffer, pH 9.6 overnight at 4°C. For immunofluorescence detection, MCF-10A neoT cells were cultured on either fibronectin-coated or uncoated coverslips for 24 hours. Cells were fixed with 2% paraformaldehyde at room temperature for 40 minutes, then permeabilized with 0.05% Triton-X 100. To preserve membrane associated components and foreclose cytoplasmic staining, some samples were not permeabilized with Triton-X 100. Non-specific staining was blocked with 3% BSA in phosphate buffer saline (PBS), pH 7.4, for 1 hour. After 1.5 hour of incubation with anti-CK MAb and either rabbit anti-uPA polyclonal antibody or goat anti-plasmin(ogen) polyclonal antibody (Santa Cruz Biotechnology, CA), cells were washed with PBS. Secondary Alexa-488 labeled anti-rabbit or Alexa-488 labeled anti-goat and Alexa-555 labeled anti-mouse antibody were incubated for 1 hour in blocking buffer and afterwards washed with PBS. Actin was labeled with phalloidin-tetramethylrhodamine B isothiocyanate conjugate (Fluka) (500 ng/mL) for 30 min at room temperature in anti-CK MAb treated or un-treated cells, then washed with PBS. Prolong Antifade kit (Molecular Probes, CA) was used for mounting coverslips on glass slides. Fluorescence microscopy was performed using a Carl Zeiss LSM 510 confocal microscope. Alexa 488 and FITC were excited with an argon (488 nm) laser and Alexa 546 with a He/Ne (543 nm) laser. Emission was filtered using narrow band 505-530 nm or LP 560 nm filters, respectively. Images were analyzed using Carl Zeiss LSM image software 3.0.

### Surface plasmon resonance

The binding of uPA to the peptide VKIALEVEIATY (Doljak et al., 2008) was monitored using a Biacore X system (Biacore, Sweden). The assay was performed on a CM5 sensor chip (BR-1003-98 BIAcore). VKIALEVEIATY was immobilized at a flow rate of 1 μL/min for 10 minutes (300 RU). The flow cell was then washed with 5 μL of 10 mM glycine buffer (pH 2.2) at a flow rate of 30 μL/min. The second flow cell was used as the reference cell. Identical wash cycles were used to regenerate the dodecapeptide surface between the assays. uPA was prepared in PBST buffer (PBS with 0.05% Tween 20). All steps were performed at 25°C with a flow rate of 1 μL/min in 1% PBST running buffer. 5 μL of uPA was injected for each assay.

### Cell invasion assay

Transwell plates (Corning Costar, MD) with 12-mm polycarbonate filters and 12 μm pore size, were used to test the effect of anti-CK MAb on invasion of breast cancer cells. 25 μl of 100 μg/mL fibronectin (Sigma, Germany) was applied on the lower side of the filters and left for one hour in a laminar hood to dry. The upper sides of the filters were then coated with 100 μL of 1 mg/mL Matrigel (Becton Dickinson, NJ), 100 μL of DMEM was added, and the filters left at 37°C for one hour. The upper compartments were then filled with 500 μL of MCF-10A neoT cells (4 × 10^5 ^cells/mL) while 1.5 mL of medium was added to the lower compartments. Anti-CK MAb (1 μM) was added to the compartments. The control cells were plated without the addition of anti-CK MAb. To determine the percentage of invasion of treated and control cells, the separate lower compartments were filled with 500 μL of MCF-10A neoT cells (4 × 10^5 ^cells/mL) and the plate incubated for 24 hours at 37°C and 5% CO_2_. The lower compartments were washed with PBS and the cells detached with 0.05% trypsin and 0.02% EDTA in PBS, pH 7.4. Cells from the lower compartments were transferred separately to Eppendorf tubes and centrifuged at 1500 rpm for 5 minutes. They were resuspended in culture medium and transferred to 96-well plates. The Cell Proliferation Assay CellTiter 96^® ^One Solution from the (3-(4,5-dimethylthiazol-2-yl)-5-(3-carboxymethoxyphenyl)-2-(4-sulfophenyl)-2H-tetrazolium) MTS (colorimetric assay, Promega, WI) was added and the plate incubated for an additional 2 hours. The absorbance, A, of the formazan product was measured at 590 nm. Invasion was recorded as the percentage of the cells that penetrated the Matrigel-coated filters:



### Cell adhesion assay

A 96-well culture plate was pre-coated with 50 μL of fibronectin (1 μg/mL) in carbonate buffer, pH 9.6, overnight at 4°. Wells were then washed once with PBS and incubated with 1% BSA in PBS for 30 min at room temperature. MCF-10A neoT cells were harvested, washed with PBS and resuspended in the serum-free medium. Anti-CK MAb was added to the medium at a final concentration 1 μM. 50 μL of MCF-10A neoT cell suspension was added to each well of a 96-well culture plate (TPP, Switzerland) pre-coated with fibronectin. Cells were allowed to attach for 15 min, 45 min, 1.5 h, 3 h and 5 h, after which wells were washed twice with PBS, and 100 μL of complete growth medium was added. The assay was performed in quadruplicate. Control wells were washed with 50 μL of medium. Finally, CellTiter 96^® ^One Solution was added, formazan absorbance measured, and cell adherence was calculated from the equation:



where the absorbance of formazan were determined for cells washed at different time points (A_tx_) and for cells not washed (A_control_).

Differential interference contrast images were taken with an Olympus IX 81 motorized inverted microscope and Cell^® ^software.

### Cell cycle analysis

A 12-well culture plate was pre-coated with 250 μL of fibronectin (1 μg/mL) in carbonate buffer, pH 9.6 overnight at 4°C. Wells were washed once with PBS and incubated with 1% BSA in PBS for 30 min at room temperature. MCF-10A neoT cells were harvested, washed with PBS and resuspended in the serum-free medium in the presence of anti-CK MAb (1 μM), while the antibody was not added to control wells. MCF-10A neoT cells (1 × 10^5 ^cells, 500 μL) were added to a 12-well culture plate (TPP) pre-coated with fibronectin. Control wells were not pre-coated with fibronectin. Cells were cultured for either 48 h or 72 h, washed twice with PBS and detached with 0.05% trypsin and 0.02% EDTA in PBS, pH 7.4. Cells were centrifuged at 1500 rpm for 5 min, resuspended in ice-cold ethanol and incubated at -20°C for 45 min. Cells were then centrifuged again and propidium iodide and RNAse added to final concentrations of 40 μg/ml and 0.1%. Cells were incubated for 30 min at 37°C, washed with PBS and analyzed for DNA content. Flow cytometry was performed on a FACS Calibur (Becton Dickinson, NJ).

### Cell proliferation assay

A 12-well culture plate was pre-coated overnight with 250 μL of fibronectin in carbonate buffer, pH 9.6 (1 μg/mL), at 4 °C. Wells were washed once with PBS and incubated with 1% BSA in PBS for 30 min at room temperature. MCF-10A neoT cells were harvested, washed with PBS, labeled with CFSE (5-carboxyflourescein diacetate succinimidyl ester) cell tracing reagent (Molecular Probes, CA) according to the manufacturer's protocol, and re-suspended in the serum-free medium, with or without anti-CK MAb (1 μM). MCF-10A neoT cells (1 × 10^5 ^cells, 500 μL) were added to a 12-well culture plate (TPP) pre-coated with fibronectin. Control wells were not pre-coated with fibronectin. Cells were cultured for either 48 h or 72 h. Wells were then washed twice with PBS and cells detached with 0.05% trypsin and 0.02% EDTA in PBS, pH 7.4. The threshold for proliferating cells was set according to MCF-10A neoT cells measured for CFSE fluorescence intensity at the beginning of the assay. Flow cytometry was performed on a FACS Calibur (Becton Dickinson).

### Statistical analysis

SPSS PC software package (Release 13.0) was used for statistical analysis. The difference between the groups was evaluated using the non-parametric Mann-Whitney test. P values of < 0.05 were considered to denote statistical significance.

## Competing interests

The authors declare that they have no competing interests.

## Authors' contributions

NO conceived the study and participated in its design, performed the experiments and drafted the manuscript. BD performed the experiments. JK participated in the coordination, supervised the study and helped to draft the manuscript. All authors read and approved the final manuscript.

## Supplementary Material

Additional file 1 **S1 - Competition of anti-CK MAb and uPA for binding to immobilized peptide VKIALEVEIATY**. Graph showing the competition of anti-CK MAb and uPA for binding to immobilized peptide VKIALEVEIATY. The concentration of anti-CK MAb was 6.7 nM. Secondary goat-antimouse antibody conjugated to HRP was used for the detection of bound anti-CK MAb. Click here for file

Additional file 2 **S2 - Adhesion of MCF-10A neoT cells to fibronectin in the absence of anti-CK MAb**. Video showing adhesion of MCF-10A neoT cells to fibronectin in the absence of anti-CK MAb. Click here for file

Additional file 3 **S3 - Adhesion of MCF-10A neoT cells to fibronectin in the presence of anti-CK MAb**. Video showing the adhesion of MCF-10A neoT cells to fibronectin in the presence of anti-CK MAb. Click here for file
