# Supplementary figures and images for: Cytokeratin 8 ectoplasmic domain binds urokinase-type plasminogen activator to breast tumor cells and modulates their adhesion, growth and invasiveness
Source: Mol Cancer. 2009 Oct 21;8:88. doi: 10.1186/1476-4598-8-88 (PMC2774675; doi:10.1186/1476-4598-8-88)

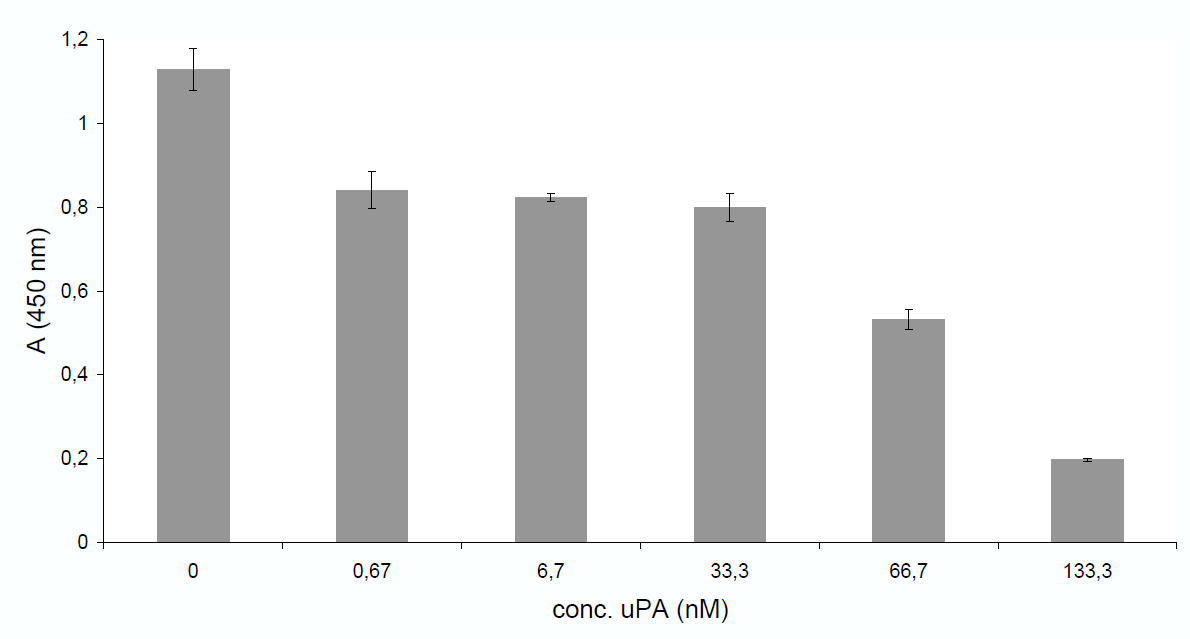

Supplement: Additional file 1 — S1 - Competition of anti-CK MAb and uPA for binding to immobilized peptide VKIALEVEIATY. Graph showing the competition of anti-CK MAb and uPA for binding to immobilized peptide VKIALEVEIATY. The concentration of anti-CK MAb was 6.7 nM. Secondary goat-antimouse antibody conjugated to HRP was used for the detection of bound anti-CK MAb. [file 1476-4598-8-88-S1.TIFF]
